# Ricin Detection Using Phage Displayed Single Domain Antibodies

**DOI:** 10.3390/s90100542

**Published:** 2009-01-19

**Authors:** Ellen R. Goldman, Jinny L. Liu, Rachael D. Bernstein, Marla D. Swain, Stanley Q. Mitchell, George P. Anderson

**Affiliations:** 1 Naval Research Laboratory, Center for Bio/Molecular Science and Engineering, 4555 Overlook Ave SW, Washington DC 20375, USA; 2 Nova Research Inc, 1900 Elkin Street, Suite 230, Alexandria VA 22308 USA; 3 Clark Atlanta University, Department of Biological Sciences, Atlanta GA 30314 USA

**Keywords:** Ricin, single domain antibody, phage, Luminex, phage display

## Abstract

Phage-displayed single domain antibodies (sdAb) were compared to monomeric solubly expressed sdAb and llama polyclonal antibodies for the detection of ricin. SdAb are comprised of the variable domain derived from camelid heavy chain only antibodies (HcAb). Although HcAb lack variable light chains, they as well as their derivative sdAb are able to bind antigens with high affinity. The small size of sdAb (∼16 kDa), while advantageous in many respects, limits the number of labels that can be incorporated. The ability to incorporate multiple labels is a beneficial attribute for reporter elements. Opportunely, sdAb are often selected using phage display methodology. Using sdAb displayed on bacteriophage M13 as the reporter element gives the potential for incorporating a very high number of labels. We have demonstrated the use of both sdAb and phage- displayed sdAb for the detection of ricin using both enzyme linked immunosorbent assays (ELISAs) and Luminex fluid array assays. The phage-displayed sdAb led to five to ten fold better detection of ricin in both the ELISA and Luminex assays, resulting in limits of detection of 1 ng/mL and 64 pg/mL respectively. The phage-displayed sdAb were also dramatically more effective for the visualization of binding to target in nitrocellulose dot blot assays, a method frequently used for epitope mapping.

## 1. Introduction

Ricin is a potent toxin derived from the beans of the castor bean plant, *Ricinus communis*. Due to the worldwide production of castor oil, large quantities of the byproduct toxin are obtainable. Isolation of the toxin is simple and inexpensive, making the sensitive detection of ricin a high priority. A number of immunoassays have been reported for the detection of ricin including electrochemiluminescence, surface plasmon resonance, fiber optic, as well as hand-held and array based immunoassays [1–6]. Immunoassays commonly rely on monoclonal or polyclonal antibodies (IgG) derived from mice, rabbits, goats or sheep as recognition elements and are easily performed, sensitive, and specific. However the sensitivity of any immunoassay is fundamentally determined by both the affinity of the specific antibodies used in the assay and the method used for signal generation.

**Figure 1.**
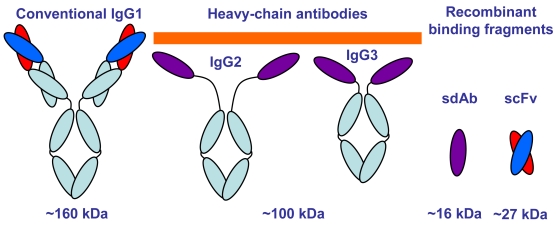
Cartoon showing conventional antibodies, heavy chain antibodies, and their cloned binding elements. Variable domains have been colored red and blue for the conventional VH and VL respectively, and purple for the VHH.

Conventional IgG antibodies are made up of two identical heavy chains and two identical light chains, with the antigen binding regions formed from a variable domain from the heavy chain (VH) paired with a variable domain from the light chain (VL). Recombinant binding elements, single chain antibodies (scFv), can be constructed by joining the VH and VL with a flexible linker [7, 8]. Members of the Camelide family, including camels, llamas and alpaca, possess both conventional IgG (IgG1) as well as unconventional IgG subclasses (IgG2 and IgG3) that consist of only two heavy chains (Figure 1) [9, 10]. The heavy chain antibody's two antigen-binding sites are each formed by only a single variable domain (VHH). These VHH can be expressed recombinantly, and have been termed single domain antibodies (sdAb) [11]. There are several advantages for using sdAb over scFv, including enhanced stability of sdAb demonstrated by their ability to refold after heat or chemical denaturation [12–14].

SdAb genes can be cloned from mRNA of lymphocytes isolated from either unimmunized llamas or llamas immunized with the antigens of interest to generate either non-immune [15–17] or immune [18-21] libraries, respectively. A display methodology, such as phage display is then used to isolate antigen binding clones from the background of non-binders [22, 23]. We recently selected ricin binding clones from an immune llama-derived library of sdAb displayed on bacteriophage M13. Many of the sdAb binders isolated from the library were demonstrated to be specific for ricin, able to re-fold after heat denaturation, and to enable detection of the toxin down to at least 1.6 ng/mL [24]. In the current work we have evaluated both the soluble sdAb as well as the phage-displayed sdAb as reporter elements in sandwich immunoassays using enzyme linked immunosorbent assays (ELISAs) and the Luminex flow analyzer. The Luminex is a flow cytometer-based instrument, which can evaluate sandwich immunoassays performed on the surface of color coated microspheres. The instrument facilitates multiplexed assays through the availability of 100 different microsphere sets. In both formats the phage-displayed sdAb provide signal amplification over the soluble sdAb.

## 2. Results and Discussion

Recently, we constructed a phage display library of sdAb, derived from IgG type 2 llama heavy chain antibody. To construct this library mRNA was isolated from the peripheral blood lymphocytes obtained from two llamas immunized with ricin toxoid [24]. Before starting library construction we evaluated the polyclonal response, and also sub-fractionated the polyclonal IgG into both conventional and heavy chain antibody fractions to ensure there was a population of high titer heavy chain antibodies that recognized ricin, similar to work described in [25].

Three rounds of selection on ricin were preformed, isolating specific binding clones after each round. The sdAb isolated had binding constants that ranged from 0.04 nM to 500 nM. Using the isolated sdAb in pairs for sandwich immunoassays, we found that they bound to at least three different epitopes on ricin. Two sdAbs binding to different epitopes on ricin are required to perform a sandwich assay. During the course of this work we determined that clones C8 and F11, when paired as detector and capture sdAb, respectively, provided excellent ricin detection sensitivity [24]. Herein, we examined the use of soluble C8 sdAb, phage-displayed C8, and llama polyclonal anti-ricin antibody for the detection of ricin captured by either F11 sdAb or llama polyclonal antibody immobilized in microtiter plate wells or on the surface of microspheres.

### 2.1. Evaluation of biotinylation reagents

**Figure 2.**
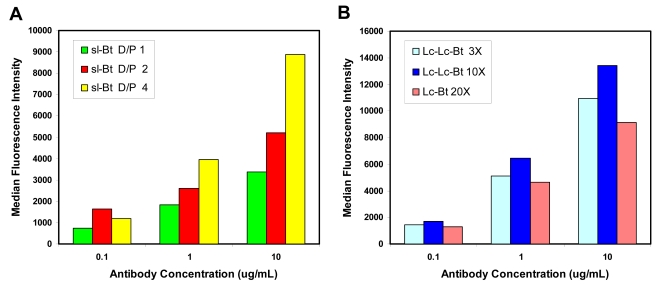
Evaluating the role of biotinylation extent or linker length. Various biotinylated llama anti-ricin IgG were examined in a direct binding assay to ricin A chain-coated microspheres; Panel A shows signal with increasing number of biotins per antibody (D/P) as determined using sureLINK biotin, while Panel B shows labeling with Lc and Lc-Lc biotin at different ratios of biotin-NHS to antibody. Results indicated that high biotin to protein ratios and longer linker lengths produce superior reagents.

We evaluated biotinylated reporter elements in both the ELISA and Luminex based assays. Llama anti-ricin was biotinylated with three different biotin reagents, sureLINK biotin-NHS (sl-Bt), biotin-long chain NHS (Lc-Bt) and biotin long chain long chain NHS (Lc-Lc-Bt). The sureLINK biotin incorporates a chromophore along with the biotin which facilitates the easy determination of the number of labels per antibody. As the number of biotins per antibody increased, we observed higher signal in direct binding assays (Figure 2A). Examining antibody labeled with each of these reagents, we found highest signal in direct binding assays to immobilized ricin A chain when the Lc-Lc-Bt was used for labeling (Figure 2B). The longer length linker between the protein and the biotin appears to make the biotins more available to the streptavidin-phycoerthrin conjugate used to produce the binding signal. However, the number of biotins incorporated in this labeling was not determined, making this supposition tenuous. Preliminary experiments were conducted using the sl-Bt to estimate the number of biotins incorporated with both the soluble sdAb (6 per sdAb) and phage-displayed sdAb (121 per phage-displayed sdAb), however as the Lc-Lc-Bt reagent provided more robust signals it was used for most of the subsequent assays.

### 2.2. ELISA using soluble sdAb and phage-displayed sdAb

**Figure 3.**
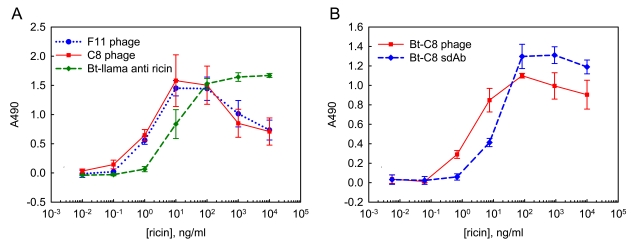
ELISA based ricin detection. Phage-displayed sdAb and llama polyclonal anti-ricin IgG were compared as the reporter reagents using a llama anti-ricin capture surface. Panel A shows results of phage-displayed sdAb followed with an anti-M13 –HRP conjugate for color generation and biotinylated llama anti-ricin IgG followed with a Streptavidin – HRP conjugate. Panel B show the results using biotinylated phage-displayed sdAb and biotinylated soluble sdAb reporter elements followed with a Streptavidin –HRP conjugate. Data is plotted as the average of 3 wells, with error bars representing the standard deviation. Results indicate phage-displayed sdAb can act as highly sensitive detection reagents.

ELISA was used for initial experiments investigating the utility of phage-displayed sdAb in sandwich assays for ricin detection. In the first tests, phage-displayed sdAb reporter elements were utilized in combination with a llama polyclonal antibody capture surface. We compared phage-displayed clones C8 and F11 to a biotinylated llama polyclonal antibody. Both the capture and reporter llama polyclonal antibodies consisted of a purified polyclonal antibody mixture containing conventional and heavy chain classes of llama IgG. Signal from the phage was generated using an anti-phage HRP conjugate while signal from the conventional antibody was generated with streptavidin HRP. Results are shown in Figure 3A; this data is representative of several ELISAs. The phage-displayed sdAb performed better than the biotinylated polyclonal llama antibody in these assays. We regularly achieved detection of at least 1 ng/mL ricin, using the phage-displayed sdAb.

After showing that the phage-displayed sdAb led to sensitive ricin detection we compared their use to biotinylated soluble sdAb recognition elements. In these sets of experiments biotinylated phage was used in conjunction with streptavidin HRP to generate signal. Figure 3B, shows the results of these experiments; again more sensitive detection was found using the phage-displayed sdAb reporter.

### 2.3. Luminex assays using soluble sdAb and phage-displayed sdAb

**Figure 4.**
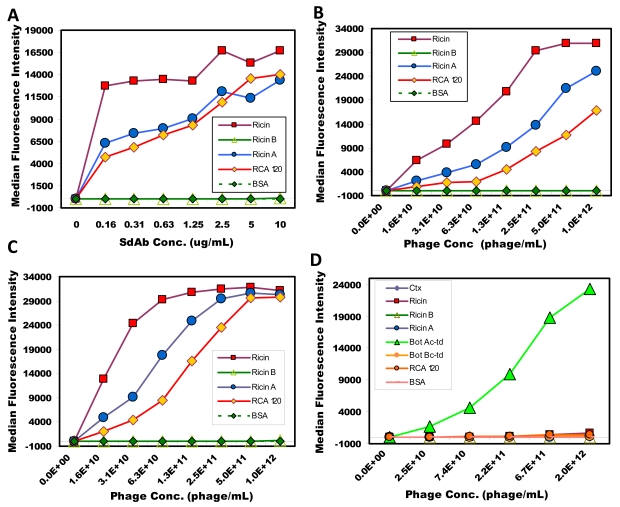
Binding of soluble and phage-displayed C8 to antigen coated microspheres. The binding of various biotinylated tracer reagents was evaluated: (A) sl-Bt-C8 sdAb, (B) sl-Bt-C8 phage, (C) Bt-Lc-Lc-C8 phage. As a control the binding of phage-displayed sdAb A4, a clone known to be specific toward botulinum toxid A complex (Bot Ac-td) is shown, (D) Bt-Lc-Lc-A4 phage. The molar concentration of soluble C8 (MW ∼ 16,000 kDa) used in these experiments varied from 10 nM to 625 nM. Molar concentration of phage varied from to 27 pM to 1.7 nM. Results indicate phage-displayed sdAb bind with high specificity toward their target antigen, with minimal nonspecific binding observed.

This work was expanded to investigate the use of phage-displayed sdAb as reporter elements for sandwich assays using the Luminex system. First, we utilized the multiplexing capabilities of the system to evaluate the binding specificity of the phage-displayed sdAb. Using a variety of toxin/protein coated microspheres, dilutions of biotinylated soluble sdAb C8 and biotinylated phage-displayed C8, both specific for ricin, as well as biotinylated phage-displayed A4, a sdAb specific for botulinum A toxoid complex, were tested. After washing away unbound phage, streptavidin phycoerythrin conjugate (SA-PE) was added and the binding evaluated. The results shown in Figure 4 indicate that even though phage are extremely large molecules, virtually no nonspecific binding is observed. C8 binds strongly to ricin, as well as to the ricin A chain, and less so to the highly homologous molecule *Ricinus communis* Agglutinin (RCA120). RCA120 is poorly discriminated by polyclonal antibodies [24] as it shares at least 80% sequence homology to ricin [27]; while C8 does show binding to RCA 120, it is significantly reduced, particularly for the phage-displayed sdAb. Meanwhile, phage-displayed A4, a sdAb known to bind botulinum A toxoid complex [19] was found to bind only its target. Phage, in addition to acting as highly specific tracers, also generated very robust signals, 5,000 units greater than observed with antibody based assays.

**Figure 5.**
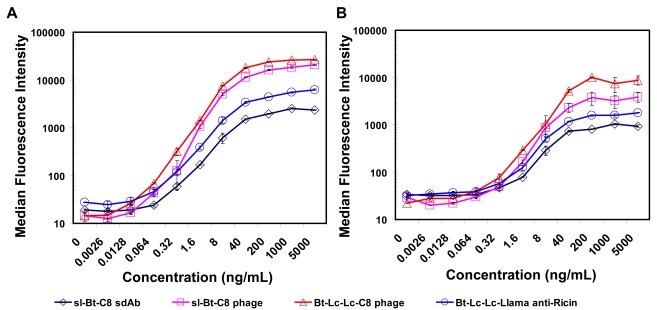
Luminex detection of ricin using llama polyclonal anti-ricin IgG (panel A) and sdAb F11 (panel B) conjugated to polystyrene beads. The data is plotted as the median fluorescence intensity with the error bars representing the range between duplicate analyses. Results show the enhanced signal generated by using the phage-displayed sdAb as the biotinylated tracer reagent relative to soluble sdAb or conventional antibody.

Sandwich assays for ricin were then performed using both llama IgG1 functionalized capture microspheres as well as with sdAb F11 functionalized capture microspheres. In these experiments sl-Bt-C8 sdAb, sl-Bt phage-displayed C8, Lc-Lc-Bt phage-displayed C8 and Lc-Lc-Bt llama anti-ricin were used as reporters followed by addition of a SA-PE. Results for the sandwich immunoassays are shown in Figure 5. When using the llama polyclonal IgG1 antibody as the capture molecule both the polyclonal and soluble sdAb tracer reagents could detect 320 pg/mL of ricin, while the phage-displayed C8 sdAb was five times more sensitive, detecting as low as 64 pg/mL. Similar results were observed on the microspheres coated with F11 sdAb, where the best phage-displayed C8 tracer could detect 320 pg/mL, approximately five-fold better than the antibody or soluble C8. These experiments were repeated twice giving nearly identical results. Because of the ability to multiplex Luminex assays, a number of additional capture surfaces including monoclonal and rabbit polyclonal anti-ricin were also examined (not shown). In all cases the highest signal was observed with the phage-displayed C8 reporter reagent, while virtual no signal was obtained from a set of negative control microsphere coated with BSA.

### 2.4. Phage as reporter reagents

Our results are in agreement with other researchers who have also obtained sensitive ELISA detection using phage-displayed antibody fragments rather than the soluble antibody fragments [28, 29]. For example, using phage sdAb reagents in ELISAs led to the detection of ∼1 pfu of Marburg virus, while use of a sdAb-alkaline phosphatase fusion led to the detection of ∼10 pfu [29]. Because of the small size of the sdAb, the sites available for modification (i.e. biotinylation) are limited. In contrast, phage-displayed reporter elements have the advantage of being very large. M13 phage has a length of 900 nm but a width of only 8 nm; its length is made up of ∼2,700 copies of pVIII, the major phage coat protein, which has a 5 kDa molecular weight. SdAb are expressed attached to one of five copies of pIII, one of the minor coat proteins found on one of the phage's ends [30, 31]. Thus, there is the potential for the attachment of many more biotin per phage than per sdAb and hence the binding of more streptavidin-HRP or SA-PE, resulting in an amplified signal. The anti-phage HRP used in the first experiments is directed against the pVIII and again the potential exists for multiple anti-phage HRP to bind on each phage detection reagent.

**Figure 6.**
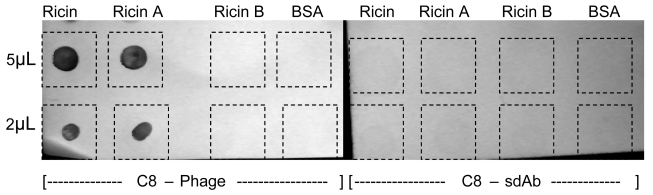
Dot blot assay for ricin, ricin A chain, and ricin B chain using C8-phage (left) and C8-sdAb (right). C8-phage binding was visualized using HRP labeled anti M13 IgG while the C8-sdAb was visualized using HRP labeled anti-His. Color formation was generated using sigmafast Diaminobenzidine (DAB). 5 or 2 μL of ricin, ricinA, ricin B or BSA (10 μg/ mL) were spotted onto nitrocellulose as the target for both phage-displayed and soluble sdAb.

In addition to their use in ELISAs, phage-displayed scFv have been used for the imaging of *Bacillus subtilis* spores [32]. In that work, phage were dye labeled and as seen for ELISAs, the multiple copies of pVIII per phage offered an advantage over dye-labeled conventional antibodies in their visualization of single spores. We have also found that the C8 displayed phage were dramatically superior for the visualization of dot blot assays for ricin. Figure 6 (left side) shows intense binding of C8-phage to both ricin and ricin A chain, but virtually no binding to ricin B chain or BSA. Corresponding experiments using soluble C8 generated much weaker signal to any of the immobilized targets (Figure 6, right side).

Phage display is often used for the selection of short peptides specific for target antigens. In these experiments, using the phage-display reagent gives the added advantage that often multiple copies of each peptide are displayed per phage. Previously, we studied the use of peptides displayed on phage as reagents for the detection of staphylococcal enterotoxin B [33]. Sandwich assays were performed with dye-labeled phage; however the selected peptides did not offer as sensitive detection as conventional antibodies. In our current work, we also tested C8-sdAb and C8-phage labeled with the fluorophore DyLight 549, however neither produced signals comparable to the same material biotinylated and thus were not pursued further. Others have used labeled phage-displayed peptides for imaging of tumor cells [34], which may lead to a valuable reagent for tumor imaging *in vivo*.

Other types of bacteriophage and virus can be employed as a mechanism for signal amplification. For example, proteins and peptides can be expressed on T4 and T7 phage [35, 36] that could be similarly labeled with biotin or fluorophores. In another example Cowpea mosaic virus capsid, with > 40 flourophors attached has been used to amplify signal on DNA microarrays and immunoassays [37, 38].

We have shown the amplification afforded using phage-displayed sdAb versus soluble monomeric sdAb as recognition elements for the detection of ricin. Phage-displayed antibody fragments have also been employed as capture elements for the detection of listeria in Surface Plasmon Resonance-based assays [39]. The ready availability of phage-displayed sdAb allows the advantages afforded by phage-based reagents to be considered in conjunction with the stability of sdAb in order to combine reagents for the production of the most effective assays for a given platform and sample type. As sdAb are often selected in a phage-display system, it is straight forward to incorporate phage reagents for target detection while also examining assays using the soluble sdAb reagent [29]. In cases where thermal stability of the reagents is more important than absolute LOD the soluble sdAb may provide the better recognition element.

## 3. Experimental Section

### 3.1. Reagents

Ricin, ricin A chain, ricin B chain, and RCA 120 were purchased from Vector (Burlingame, CA). Llama polyclonal anti-ricin was purified from serum isolated from two animals that had undergone immunizations with ricin toxoid at Triple J Farms (Bellingham, WA). PhycoLink® Streptavidin-R-Phycoerythrin (SA-PE) and Streptavidin-HRP were purchased from Prozyme (San Leandro, CA). Phosphate buffered saline (PBS), Tween 20, Sigma fast OPD, and bovine serum albumin (BSA) were obtained from Sigma-Aldrich (St. Louis, MO). The Anti-M13 HRP conjugate was purchased from GE Healthcare (Piscataway, NJ).

### 3.2. Biotinylation and fluorphore labeling of sdAb, polyclonal antibodies, and phage

The reagents were biotinylated with either sl-Bt-NHS from KPL (Gaithersburg, MD) or NHS-Lc-Bt or NHS-Lc-Lc-Bt from Pierce (Rockford, IL). The various biotins were dissolved in either DMSO or DMF and added to the antibodies at a ratio of 10:1 for most applications. Phage were biotinylated by reacting 200 μL (∼10^13^ virons/mL) of phage stock with (0.3 mg/mL) of the various NHS-Bts. For the IgGs the excess biotin was removed using a Bio-gel P10 column (BioRad, Hercules, CA), while free biotin was removed from the sdAb and phage by dialysis using Slide-A-Lyzer MINI dialysis units from Pierce. Using the sureLink biotin we obtained 6 biotin per sdAb and 121 biotins per phage. The number of biotin per sdAb and phage were calculated per the manufacturer's instructions. C8 sdAb and phage were also fluorphore labeled using DyLight 549 NHS (Pierce). Excess dye was removed by dialysis. The C8 sdAb was labeled with a dye/protein ratio of 4, while the C8 phage had an approximate dye/phage ratio of ∼2,000.

### 33. Preparation of soluble and phage-displayed sdAb reagents

Phage were prepared according to standard protocols. Briefly, sdAb clones in the phage-display vector were grown in *E. coli* XL1 blue (Stratagene, La Jolla, CA) to exponential phase at 37°C in LB (100 μg/mL ampicillin and 2% glucose), and rescued with M13KO7 helper phage (New England Biolabs, Beverly MA). Infected cells were grown overnight in LB (100 μg/mL ampicillin and 30 μg/mL kanamycin) at 30°C. Phage were purified by polyethylene glycol/NaCl precipitation and resuspended in a total volume of 2 mL PBS. Phage concentration was determined from the absorbance at 269 nm [40], using equation 1. Using our phage-display vectors, the number of base pairs per viron is about 7969 for C8 and F11. We calculated concentrations of virons for C8 and F11 at 10^13^ virons/mL:

(1) $$\text{Virons}/\text{mL}={\left({\text{A}}_{269}-{\text{A}}_{320}\right)}^{*}6\times 10^{16}/(\text{number of bases per viron})$$

To prepare soluble sdAb, the coding sequence of the sdAb was cloned into an expression vector and transformed into *E. coli* Rosetta (Novagen, Madison, WI) for protein production. As described previously [17], the sdAb proteins were isolated from the periplasmic compartment of 500 mL scale shake flask cultures by osmotic shocking, IMAC and gel filtration on a Superdex G75 column (GE-Healthcare). Proteins were quantified using micro-BCA assay (Pierce, Rockford, IL) and stored at 4 °C prior to analysis.

### 3.4. ELISA assay protocols

Wells of 96-well plates (maxisorb, nunc) were coated overnight at 4°C with 10 μg/mL llama polyclonal anti-ricin in PBS. Wells were washed 2 times with PBS containing 0.05% tween-20 (PBST) and blocked for an hour at room temperature with PBS containing 2% weight/volume non-fat powdered milk (PBSM). After blocking wells were washed twice with PBST and ricin, diluted in PBS, added to rows A through G; row H contained PBS only. After incubating with the ricin dilutions for about an hour the wells were washed 3 times with PBST. Reporter reagents were added in the following concentrations: phage-displayed sdAb, 1% in PBS (∼10^11^ virons/mL); biotinylated llama anti-ricin, 10 μg/mL in PBS; biotinylated sdAb, 7 μg/mL in PBS; biotinylated phage, 3% in PBS (∼3×10^11^ virons/mL). Wells were incubated an hour with the reporter elements and then washed 4 times with PBST. Either anti-M13-HRP or streptavidin HRP was incubated in the wells for 45 minutes at room temperature. Wells were washed 4 times and signal developed with sigmafast OPD. After about five minutes 4M H_2_SO_4_ was added to stop the color development and the absorbance at 490 was read using a Tecan Saphire plate reader.

### 3.5. Preparation of Luminex reagents and assay protocols

Luminex (Austin, TX) carboxylated microspheres were cross linked to a variety of proteins using the two-step carbodiimide coupling protocol provided by the manufacturer. The signal for Luminex experiments is reported as the median fluorescence intensity of at least 100 separate microspheres.

For direct binding assays biotinylated tracer reagents were serially diluted in a 96-well microtiter plate (60 μL/well). To each well a mixture of protein coated microspheres, including spheres coated with ricin, ricin A chain, ricin B chain and RCA120, was added (5 μL/well). The microspheres and Bt-tracers were allowed to incubate for at least 30 minutes at room temperature. Unbound Bt-tracer was removed by filtration, then 5 μL/well (10 mg/L) of SA-PE was added and incubated for an additional 30 minutes. After the excess was removed by filtration the microsphere were resuspended in 85 μl PBST and transferred to standard microtiter plate prior to measuring using the Luminex 100 flow analyzer.

For the sandwich immunoassays, selected antibody-coated microspheres were incubated (30 minutes, room temperature), in wells of a 1.2 μm multiscreen filter plate (Millipore, Billerica, MA) with different amounts ricin. Excess ricin was removed by filtration, and Bt-tracer added; antibodies at 10 mg/L, sdAb at 5 mg/L, and phage at 5% (∼5×10^11^ virons/mL) of the stock. After 30 minutes of incubation (room temperature) the excess Bt-tracer was removed by filtration and then SA-PE (5 mg/L) was added and the plate incubated at room temperature in the dark for 30 minutes. Binding was then evaluated using the Luminex instrument.

## 4. Conclusions

Using phage-displayed single domain antibodies as reporter reagents, we were able to detect ricin down to levels five to ten-fold lower than using either polyclonal reagents or monomeric, soluble sdAb. The simple and convenient path to signal amplification provided by the phage should greatly improve the utility of antibody fragments for a broad array of biosensor applications.
